# The mechanisms of crystal growth inhibition by organic and inorganic inhibitors

**DOI:** 10.1038/s41467-018-04022-0

**Published:** 2018-04-20

**Authors:** S. Dobberschütz, M. R. Nielsen, K. K. Sand, R. Civioc, N. Bovet, S. L. S. Stipp, M. P. Andersson

**Affiliations:** Nano-Science Center, Department of Chemistry, University of Copenhagen, Universitetsparken 5, DK-2100 København Ø, Denmark

## Abstract

Understanding mineral growth mechanism is a key to understanding biomineralisation, fossilisation and diagenesis. The presence of trace compounds affect the growth and dissolution rates and the form of the crystals produced. Organisms use ions and organic molecules to control the growth of hard parts by inhibition and enhancement. Calcite growth in the presence of Mg^2+^ is a good example. Its inhibiting role in biomineralisation is well known, but the controlling mechanisms are still debated. Here, we use a microkinetic model for a series of inorganic and organic inhibitors of calcite growth. With one, single, nonempirical parameter per inhibitor, i.e. its adsorption energy, we can quantitatively reproduce the experimental data and unambiguously establish the inhibition mechanism(s) for each inhibitor. Our results provide molecular scale insight into the processes of crystal growth and biomineralisation, and open the door for logical design of mineral growth inhibitors through computational methods.

## Abstract (web summary)

Although trace compounds are known to inhibit crystal growth, the mechanisms by which they do so are unclear. Here, the authors use a microkinetic model to study the mechanisms of several inhibitors of calcite growth, finding that the processes are quite different for inorganic and organic inhibitors.

## Introduction

The ability to predict the behaviour during crystal growth would have an important scientific and economic impact. Accurate prediction of mineral growth rates in complex fluids is the base for understanding a broad range of geological processes, from crystallisation of a melt in a magma chamber, through recrystallisation during diagenesis and metamorphism, to the production of secondary minerals during weathering, and the controls on biomineralisation. From an applied perspective, it would provide the ability to design biomimetic materials with predetermined properties^1,2^ (i.e. high mechanical strength and controlled surface area), it would aid in the systematic improvement of techniques to prevent or remove unwanted precipitation such as scale formation in pipes^3^, and it would provide input for the improved manufacture of crystalline materials.

Over past decades, several models for mineral growth in the presence of inhibitors have been developed. Most require several free parameters and some empirical assumptions for deriving the model equations^4,5^. The classic Cabrera–Vermilyea model (CV model)^4^ focusses on blocking the step edge advance. A different approach, by Nielsen et al.^5^, expanded on the kinetic ionic model for a Kossel crystal, presented by Zhang and Nancollas^6^, and described inhibition as a result of either kink-site blocking, the kink-blocking model, or incorporation of the inhibitor into the crystal, the incorporation inhibition model. These models are described in more detail in Supplementary Note 1. A recently published microkinetic model for calcite growth^7^ uses only one physically meaningful parameter of each inhibitor, namely the adsorption energy of the inhibitor on the crystal step. The thermodynamic base and the absence of empirical parameters makes the microkinetic model simple, yet enables reliable predictions and straightforward interpretations. The microkinetic model was developed for minerals consisting of two general components in equal stoichiometry, and should therefore be generally applicable to the growth of any two components of a crystal, also at elevated temperature and pressure. This would, for example, include the most divalent metal carbonate and sulphate minerals, as long as kink nucleation is observed to be a rate-limiting step during growth.

Calcite is an important link in the global carbon cycle, it is present in nearly all the earth’s surface environments and it plays a leading role in biomineralisation^8–10^, where the organic molecules have been shown to significantly impact the growth rate and morphology of calcite^11–13^. Currently, our interest focuses on the structures and properties of biocomposites^14^, internal cell structure in relation to the mineralising compartment^15^ and the role of the polymers for inducing mineralisation^16–18^.

In this work, we extend the microkinetic model^7^ for calcite growth to accommodate adsorption of foreign species. Our goal was to understand the mechanisms that control crystal growth when ionic or organic inhibitors are present. We test the extended microkinetic model on calcite growth at ambient temperature in aqueous solutions containing ionic and small organic inhibitors. Magnesium is a known poison for calcite growth, and this is relevant for biomineralisation^19–21^ as well as industrial materials synthesis^22^, so we test the model with the recently published^23^ experimental data for magnesium (Mg^2+^), sulphate (SO_4_^2−^) and their ion pair (MgSO_4_^0^). Organic compounds, in the form of humic and fulvic acids, are common in the earth’s surface waters, and the carboxyl functional group is also active in molecules known to control calcite growth^11^ and biomineralisation^12^, so we test the model with new experimental data for calcite growth in solutions containing acetate (CH_3_COO^−^) and benzoate (C_6_H_5_COO^−^). Our results show that inhibition by the inorganic species Mg^2+^ and SO_4_^2−^ is caused mainly by adsorption on calcite steps, which blocks kink nucleation. However, inhibition by the small organic acids mainly results from complexing in the solution, which decreases the number of growth units available for attachment on the surface. We demonstrate that the minimal set of parameters used in the microkinetic model together with geochemical speciation modelling is enough to reproduce the observed behaviour for all inorganic and organic inhibitors in our study.

## Results

### Inhibition by the inorganic ions Mg^2+^ and SO_4_^2−^

We began by modelling the effect of inorganic inhibitors, with Mg^2+^ and SO_4_^2−^ as examples. To include these species in the microkinetic growth model^7^ in a thermodynamically consistent manner requires the knowledge of the solution speciation. The formation of ion pairs has been suggested as a potential growth inhibition mechanism^24^, significantly affecting growth by decreasing the saturation index (SI). We therefore performed a full geochemical speciation calculation with PHREEQC^25^ for each experimental data point. The solution speciation results for the Mg, SO_4_ and MgSO_4_ systems were used in the microkinetic model, fitted to the experimental data. The equilibrium constant for CaCO_3_^0^ ion pair formation is $K_{{\mathrm{CaCO}}_3^0} = K_{{\mathrm{IP}},{\mathrm{CaCO}}_3} = 10^{3.22}$ in the PHREEQC database^26,27^. We have used the standard geochemistry notation for the ion pair, CaCO_3_^0^. We use the IP notation to specify that it relates to the ion pair formation in the solution, which avoids confusion with adsorption equilibrium constants, which are written as *K*_X_, for adsorption of X onto the steps. Because the experimental data^23^ for MgSO_4_ inhibition were acquired at low SI (0 < SI < 1, where 0 = equilibrium), the growth from polynuclear complexes was negligible^28–30^, so we can use the simpler ion pair (IP) model^7^ to obtain microscopic growth rates (step advancement rates) in the presence of a single inhibitor (Mg^2+^ or SO_4_^2−^):

1 $${r_{{\mathrm{SO}}_4} = \frac{{a \cdot K_{{\mathrm{IP}},{\mathrm{CaCO}}_3}\left[ {{\mathrm{Ca}}^{2 + }} \right]\left[ {{\mathrm{CO}}_3^{2 - }} \right]}}{{\left( {1 + K_{{\mathrm{Ca}}}\left[ {{\mathrm{Ca}}^{2 + }} \right]} \right)\left( {1 + K_{{\mathrm{CO}}_3}\left[ {{\mathrm{CO}}_3^{2 - }} \right] + K_{{\mathrm{SO}}_4}\left[ {{\mathrm{SO}}_4^{2 - }} \right]} \right)}}\ \frac{{kT}}{h}{{\rm e}}^{ - \left( {\frac{{\Delta G_{{\mathrm{IP}},{\mathrm{CaCO}}_3}}}{{{RT}}}} \right)}\,\rm{and}}$$

2 $${r_{{\mathrm{Mg}}} = \frac{{a \cdot K_{{\mathrm{IP}},{\mathrm{CaCO}}_3}\left[ {{\mathrm{Ca}}^{2 + }} \right]\left[ {{\mathrm{CO}}_3^{2 - }} \right]}}{{\left( {1 + K_{\rm{Ca}}\left[ {{\mathrm{Ca}}^{2 + }} \right] + K_{{\mathrm{Mg}}}\left[ {{\mathrm{Mg}}^{2 + }} \right]} \right)\left( {1 + K_{{\mathrm{CO}}_3}\left[ {{\mathrm{CO}}_3^{2 - }} \right]} \right)}}\ \frac{{kT}}{h}{\rm e}^{ - \left( {\frac{{\Delta G_{{\mathrm{IP}},{\mathrm{CaCO}}_3}}}{{{RT}}}} \right)}}$$

Here, the step spacing of the atomic structure, *a*, is 0.32 nm, and $\Delta G_{{\mathrm{IP}},{\mathrm{CaCO}}_3}$ represents the energy barrier for the rate-limiting reaction, i.e. kink nucleation. It mostly reflects the energy required for dehydrating the attaching unit. $\frac{{kT}}{h}$ denotes the attempt frequency, which is equal to the product of the Boltzmann constant and temperature, divided by the Planck constant. For growth inhibition by both Mg^2+^ and SO_4_^2−^, the expressions above can be generalised assuming that the ions adsorb independently:

3 $${{r_{{\mathrm{MgSO}}_4} = \frac{{a \cdot K_{{\mathrm{IP}},{\mathrm{CaCO}}_3}\left[ {{\mathrm{Ca}}^{2 + }} \right]\left[ {{\mathrm{CO}}_3^{2 - }} \right]}}{{\left( {1 + K_{{\mathrm{Ca}}}\left[ {{\mathrm{Ca}}^{2 + }} \right] + K_{{\mathrm{Mg}}}\left[ {{\mathrm{Mg}}^{2 + }} \right]} \right)\left( {1 + K_{{\mathrm{CO}}_3}\left[ {{\mathrm{CO}}_3^{2 - }} \right] + K_{{\mathrm{SO}}_4}\left[ {{\mathrm{SO}}_4^{2 - }} \right]} \right)}}} \ \frac{{kT}}{h}{\mathrm{e}}^{ - \left( {\frac{{\Delta G_{{\mathrm{IP}},{\mathrm{CaCO}}_3}}}{{RT}}} \right)}}$$

We can then relate the microscopic growth rate, $r$, from the models to the measured inhibition index, ${\it \Theta}$, by:

4 $${\Theta} = \frac{{r_{{\mathrm{uninhib}}} - r_{{\mathrm{inhib}}}}}{{r_{{\mathrm{uninhib}}}}}$$

where *r*_inhib_ represents any of the rates from Equations (1)–(3) (details in Supplementary Note 2) and *r*_uninhib_ can be obtained by setting the inhibitor concentration to 0 in Equations (1)–(3). *r*_uninhib_ in this case reduces to the rate, *r*_IP_. We do not make any assumptions about the ratio of acute/obtuse steps in our fits, because these data are not available in a macroscopic measurement. This means that if growth is completely inhibited on one step type, but not the other, the fitted adsorption energy values only reflect the growing step, which would dominate the macroscopic growth rate. In the case of strong inhibition on one type of step and a weak inhibition on the other type of step, the fitted adsorption energy would describe the weak inhibition. Only by fitting the model to step-specific data, can the inhibition on each type of step be obtained.

**Fig. 1:**
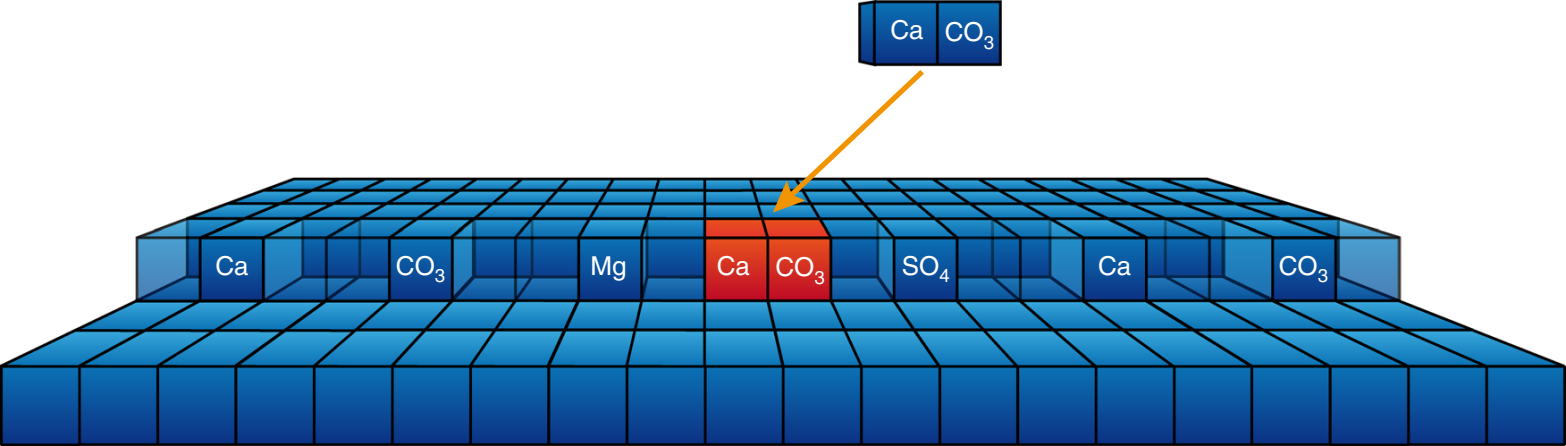
The growth inhibition model. A generic step edge consisting of empty sites (transparent) and the sites occupied by various ions present in the solution. On calcite, kinks are formed by the attachment of a calcium carbonate ion pair (in red), which is assumed to be the rate determining reaction in the growth process. Adsorbed single ions block the sites for growth unit adsorption

We fit the model in Equations (1)–(3) to the measured inhibition index, which is constructed in such a way that the fit parameter for the energy barrier, $\Delta G_{{\mathrm{IP}},{\mathrm{CaCO}}_3},$ cancels because only the relative changes in the growth rate matter. We used $K_{{\mathrm{Ca}}} = 10^{3.236}$ and $K_{{\mathrm{CO}}_3} = 10^{3.355}$, which were calculated to be the geometric mean of the equilibrium constants for adsorption of Ca^2+^ and CO_3_^2−^ on the acute and obtuse steps^7^, obtained by fitting the experimental data in Sand et al.^31^. This leaves the adsorption equilibrium constants $K_{\rm{Mg}}$ and $K_{\rm{SO}_4}$ as the only free parameters. As solution complexing must be taken into account simultaneously, it is important to use the concentration of free ions available for adsorption in the model, in Equations (1)–(3). A schematic diagram of the model is presented in Fig. 1.

**Fig. 2:**
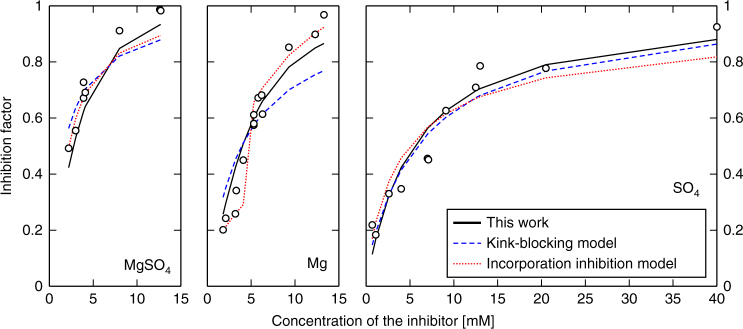
Comparison between the model and the experimental growth inhibition data for Mg^2+^ and SO_4_^2−^. Fits for the growth inhibition data with the extended microkinetic model (this work, black), the kink-blocking model (blue) and the incorporation inhibition model (red) for MgSO_4_^0^, Mg^2+^ and SO_4_^2−^. The microkinetic model fits best and requires only two physically meaningful parameters, adsorption energy for Mg^2+^ and SO_4_^2−^

Figure 2 shows the outcome of the various fitting procedures, using the parameter values listed in Supplementary Note 3 (Supplementary Tables 1-3). The data were fit with the kink-blocking and incorporation inhibition models for each inhibitor separately, using 3 and 6 free parameters per curve, as in the original models^5^. In spite of the large number of possible fitting parameters (9 and 18), the kink-blocking model (blue dashed line) underestimates the effect of MgSO_4_^0^ and Mg^2+^. The incorporation inhibition model (red dotted line) underestimates the effect of MgSO_4_^0^ and gives a fit with unphysical slope change for the Mg^2+^ data. The fit with both models is reasonable for the SO_4_^2−^ data.

The extended microkinetic growth model is able to reproduce the inhibiting effect of Mg^2+^, SO_4_^2−^ and MgSO_4_^0^. Despite using only two free parameters in total for all the three fits, it performs better than the previous models^5^; the residual is 0.15, compared with 0.26 and 0.16. Because there are no free, empirical parameters, we can interpret the results in light of the deriving growth inhibition mechanisms. The microkinetic model indicates that the effects of Mg^2+^ and SO_4_^2−^ are additive and independent of each other. Table 1 shows the derived adsorption energies of the ions. Both Mg^2+^ and SO_4_^2−^ adsorb more weakly than Ca^2+^ and CO_3_^2−^, but only by ~20 %. The results indicate that the dominant mechanism for inhibition by Mg^2+^ and SO_4_^2−^ is ion adsorption on steps, which blocks the growth sites, preventing attachment of CaCO_3_^0^ ion pairs. This is the only mechanism that affects SO_4_^2−^. For single, weakly complexing inhibitors such as SO_4_^2−^, the inhibition factor can be interpreted as the fraction of step sites covered by the inhibitor (Supplementary Note 3). For Mg^2+^, ion pairs formation in the aqueous phase contributes an additional ~10% to total inhibition at high magnesium concentration, so the solution speciation must be considered for quantitative agreement (more details in Supplementary Note 5). The results indicate that it is essential to include the solution speciation for accurate growth inhibition modelling of carbonate minerals. Equations (1)–(3) should therefore be used with concentrations or activities obtained from the PHREEQC calculations.

**Table 1:** Adsorption energies used in and obtained by the fit to experiments

Ion/inhibitor	Adsorption energy (kJ/mol)
Ca^2+^	−18.5 ± 2
CO_3_^2−^	−19.1 ± 2
Mg^2+^	−14.2 ± 0.3
SO_4_^2−^	−16.3 ± 0.3
Acetate	−1.1 ± 0.3
Benzoate	−2.8 ± 2.3

The adsorption energies and uncertainties for Ca^2+^ and CO_3_^2−^ were taken from Table S1 in Andersson et al.^7^ and averaged for the two types of calcite steps. Adsorption energies and uncertainties for Mg^2+^, SO_4_^2^, acetate and benzoate are from this work. They were obtained from the fits to the experimental data.

There is evidence that ions are incorporated into the growing crystal^23^, but the good fit of the model to the experimental growth data, without considering incorporation, suggests that incorporation is a minor or negligible growth inhibition mechanism. Substituting ions can be incorporated to form a solid solution without significant effect on the crystallisation rate. Incorporation without inhibition agrees with previous results^7^, though we observe (in Fig. 2) a small discrepancy between predictions and the experimental data. The discrepency is negligible for low inhibitor concentrations and is <5–10%, even for the highest Mg^2+^ and MgSO_4_^0^ concentrations. This small underestimation of the inhibitory effect at high Mg^2+^ concentration could result from Mg^2+^ incorporation.

While cation incorporation into calcite does not influence the growth rate significantly in itself, the subsequent change in surface composition would have a significant effect on surface energy^32,33^ and thus impact the affinity of organic inhibitors that might also be present. Divalent cation incorporation into the calcite $\left\{ {10\bar 14} \right\}$ surface has been predicted to significantly change the adsorption energy of organic molecules compared with water^32,33^. Because adsorption and step blocking is a potentially important inhibitory effect, changing the surface chemistry can significantly influence growth rate, and in more complex systems, where both cations and organic molecules are present, incorporation might need to be taken into account to describe growth.

### Inhibition by carboxylic acids acetate and benzoate

**Fig. 3:**
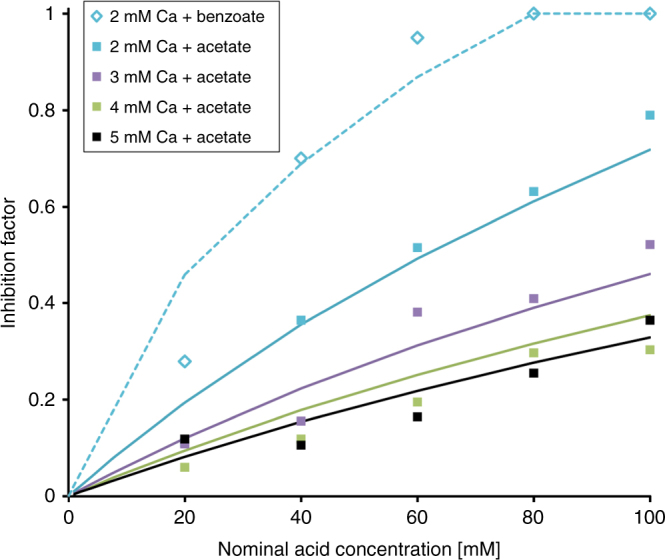
Comparison between the model and the experimental growth inhibition data for acetate and benzoate. The inhibition factor for calcite growth in the presence of the small carboxylate anions, acetate (filled squares) and benzoate (open diamonds). In the legend, *x* mM Ca refers to a starting solution of* x* mM CaCl_2_ and *x* mM NaHCO_3_

The carboxylic acids have been shown to influence calcite growth^34,35^. The inhibition mechanism has been assumed to be step blocking but this has not been proven. Therefore we made growth inhibition experiments using acetate and benzoate, two simple organic molecules containing a single carboxylate functional group. We carried out PHREEQC calculations to obtain the solution speciation for each set of conditions with varying inhibitor concentration. In our experiments, SI varied from slightly above 1 to 0. To obtain a smooth growth rate as a function of SI, we therefore used the modified IP model, where the constant growth rate is subtracted to make the net growth rate 0 at SI = 0^7^. The comparison of the experimental data with the model fit is shown in Fig. 3. The agreement of inhibition as a function of concentration was very good for both molecules, under quite different conditions. As for the inorganic ions, only one parameter per inhibitor was needed, the step adsorption energy. Because the deprotonated acids are anions, we used Eq. 1 with carboxylate concentration instead of sulphate. The optimised adsorption energy for acetate was −1 kJ/mol and for benzoate was −3 kJ/mol, which is significantly weaker than the divalent inorganic ions. The difference in the adsorption energy between acetate and benzoate from the model fit is consistent with DFT predictions, which predict that benzoic acid binds slightly more strongly than acetic acid^36^.

For the small carboxylates studied here, the change in SI and the decreased concentration of growth units is the dominating mechanism, but step blocking accounts for ~15% of the inhibition and must be considered to describe the experimental data (Fig. 3 and Supplementary Note 5). Solution complexing as the dominant inhibition mechanism is consistent with the observation that the same concentration of inhibitor is more effective for low SI conditions than for high SI. Figure 3 shows this relationship for inhibitor concentration of 100 mM. The inhibition factor drops from 0.7 to 0.3 for increasing Ca concentration (i.e. increasing SI).

## Discussion

Only the free energy from the binding of the inhibitor to the calcite step is necessary to determine the dominant calcite growth inhibition mechanism for these small organic and inorganic inhibitors. The inhibition mechanism ranges from only step blocking (SO_4_^2−^), through mainly step blocking with minor contributions from solution complexing (Mg, MgSO_4_), to mainly solution complexing with minor influence of step blocking (acetate, benzoate). Good agreement was obtained over a range of inhibitors and inhibition factors.

Solution complexing must be considered simultaneously to adsorption in order to obtain quantitative agreement between the model and the experiment. At least for the inorganic inhibitors studied here, solution complexing was less effective than blocking by adsorption, in spite of a slightly larger equilibrium constant for solution-ion pair formation (10^2.98^) than step adsorption energy (10^2.8^, derived from the fitted adsorption energy in Table 1). Inhibition via step blocking is, unsurprisingly, quite sensitive to the adsorption energy of the inhibitor (Supplementary Note 6). Magnesium incorporation, which has been suggested as one of the main inhibition mechanisms^8,37^, is predicted to account for <5–10% of the total inhibition.

With the ability to predict the extent of crystal growth and recrystallisation and the effect of inhibitors, we move closer to understanding large scale (in space and time) processes that shape our earth and its climate. With this new understanding about the molecular scale mechanisms, we come closer to understanding the processes that control biomineralisation, and with the growth inhibition model, it becomes possible to systematically design improved growth inhibitors, for example, by computational screening of complexing done by the dominant ions (in the case of calcite, for Ca^2+^or CO_3_^2^) and by determining the adsorption energies of the inhibitor species on the calcite steps. In general, strong adsorption allows for effective inhibition with lower concentrations of inhibitor.

## Methods

### Constant composition experiments

The constant composition-method setup has been used in the previous work^23,38^. Stock solutions of CaCl_2_, NaHCO_3_, Na_2_CO_3_ were prepared using reagent grade chemicals from Sigma-Aldrich. At the start of the experiment, 50 mL of 0.1 M NaCl solution containing equivalent aliquots of CaCl_2_ and NaHCO_3_ were added to target the required concentration, and the pH was adjusted to 8.3 by the addition of a few µL of 0.1 M NaOH. The 50 mL solution was introduced in a steered glass reactor vessel at room temperature (22 ± 1 ℃). When the pH was stable at 8.3 for about 30 min, the calcite seed (Merck calcite powder 99.95% Suprapure) was added to the system to initiate precipitation. When a constant growth rate was achieved, addition of inhibitors obtained from the stock solution was added. The change in calcite volume was less than 4% in the maximum concentration run. The fractional inhibition was calculated as (*R*_0_−*R*_1_)/*R*_0,_ where *R*_0_ is the initial growth rate and *R*_1_ is the growth rate after addition of the inhibitors. The PHREEQC calculations for the constant composition setup and carboxylate inhibition were conducted using the Minteq.v4 database.

### Fitting of the experimental inhibition experiments

A standard, nonlinear least squares method in the form of a trust-region reflective algorithm^39^ with a central difference scheme was used for all fits, except for the incorporation inhibition model. Because of its heavy nonlinear nature, we used simulated annealing.^40,41^ Termination tolerances were set to 1E−15 in all cases, and numerous starting values were used to ensure that the algorithms were not trapped in local optima.

### Data availability

All data are available from the authors on reasonable request

## Electronic supplementary material

Supplementary Information
