# Supplementary material for: The mechanisms of crystal growth inhibition by organic and inorganic inhibitors
Source: Nat Commun. 2018 Apr 20;9:1578. doi: 10.1038/s41467-018-04022-0 (PMC5910393; doi:10.1038/s41467-018-04022-0)
Supplement: Supplementary file 1 — Supplementary Information [file 41467_2018_4022_MOESM1_ESM.pdf]

The mechanisms of crystal growth inhibition by organic and inorganic inhibitors

Dobberschütz et al.

Supplementary Note 1

Review of growth inhibition models

For modelling growth inhibition in calcium systems, a number of mathematical models have been proposed:

- The Cabrera-Vermilyea (CV) step-pinning model[1],
- The kink blocking model[2],
- The incorporation inhibition model[2].

The CV model, proposed by Cabrera and Vermilyea (1958) as a general model, was based on observations in electrophoretic systems. It assumes that a distribution of impurities on the crystal surface hinders the advance of growing step edges. The step edges are forced to grow through or around the impurity array, thus leading to curved step segments. Because of the curvature, the Gibbs-Thomson effect plays a role and highly curved segments are hindered in their advance. Above a certain curvature limit, which corresponds to close spacing for the adsorbed impurities, step growth is no longer possible. This model is rarely applied in its original form. Potapenko[3] adjusted the step edge free energy and in that form, the model has been used by Wang et al.[4] to describe calcium oxalate monohydrate formation. Weaver and colleagues[5-7] studied the effect of citrates and peptides on calcium oxalate and adjusted the model even further, to include time dependent surface coverage by impurities.

We did not test the effectiveness of the CV model in our analysis for two reasons. The 3 fitting parameters cannot be interpreted in a physically meaningful way. Each parameter includes rate coefficients, the fraction of adsorbed impurities and geometric factors together. Recently, Lutsko and colleagues[8] made extensive MD simulations that modelled the dynamics of the processes underlying the CV model and showed that it can only be applied under very restricted conditions, which can only be fulfilled e.g. in protein mediated biomineralisation.

Nielsen et al.[2] developed two growth inhibition models based on growth theory for Kossel crystals from Zhang and Nancollas[9]. A Kossel crystal is a simple model crystal, where the growth unit is a cube with six equivalent faces, able to bind to the crystal at one or more faces. A Kossel crystal thus has steps and kinks, similar to a real crystal. The first model by Nielsen et al.[2] is valid for impurities that are not incorporated in the crystal structure but that block kink sites, whereas the second model also considers impurity incorporation. Both allow for determination of attachment and detachment rate coefficients for the various ions. A concern about these models is that the

underlying ZN model is explicitly derived for crystals consisting of systems of two species only. Adding growth units would require changing the derivation of the models, especially the "resistance to nucleation", labelled Ω in the papers. In their adaptation of the ZN model to account for site blocking or impurity incorporation, Nielsen et al.[2] did not make such adjustments. Thus, these models have a purely empirical component. All models considered in this paper work in the limit of weak inhibition, in the sense that the growth mechanism remains the same.

Supplementary Note 2

Relating microscopic and macroscopic growth rates

All the models are based on microscopic growth rate, r, where material is added to step edges only. In experiments, macroscopic rates, R, are measured in the whole system and usually include at least one of the following effects on the solid produced:

- The formation of new crystals.
- Increase in crystal surface area as growth proceeds.
- The number of crystals present in the reactor.

Because of the relatively low supersaturation in the experiment (0<SI<1), we can rule out the first factor. If the second were dominating, we would observe nonlinear growth rates, which was not the case[10]. Therefore, it is reasonable to assume that the growth rates for a single experiment are related by $R = C \cdot r$, where r represents the microscopic growth rate and C, a constant that depends on the number of seed crystals in the reaction vessel, their geometry and other factors that we assume are constant when the growth inhibitor is added. For each experiment, the inhibition index, Θ, is given by:

$$\Theta = \frac{R_{\text{uninhib}} - R_{\text{inhib}}}{R_{\text{uninhib}}} = \frac{C \cdot (r_{\text{uninhib}} - r_{\text{inhib}})}{C \cdot r_{\text{uninhib}}}. \qquad (1)$$

This allows us to express the inhibition index using microscopic growth rates only, $\Theta = \frac{r_{\text{uninhib}} - r_{\text{inhib}}}{r_{\text{uninhib}}}$, and we can fit the theoretical models to the experimental data. In cases where there is no significant complexing and the Ca^{2+} and CO_3^{2-} concentrations are unchanged when the inhibitor is added, the expression for the inhibition index takes on a particularly simple form. In our case, this applies to SO_4^{2-}. Inserting the expressions from Equations 1-3 from the main paper gives:

$$\Theta = \frac{K_{\text{SO}_4}[\text{SO}_4]}{1 + K_{\text{CO}_3}[\text{CO}_3] + K_{\text{SO}_4}[\text{SO}_4]}. \qquad (2)$$

The relationship for a single, noncomplexing inhibitor is the Langmuir isotherm expressions for competitive adsorption between the inhibitor and the Ca^{2+} or CO_3^{2-} binding to the same site. Thus, the inhibition index in such a case can be interpreted as the coverage of the inhibitor on the step sites, which allows a very intuitive interpretation of the inhibition data.

Supplementary Note 3

Fit parameters obtained for inhibition by Mg^{2+} and SO_4^{2-} by all three models

Supplementary Tables 1, 2 and 3 provide the parameters derived from fitting the microkinetic model, the kink blocking model and the incorporation inhibition model to the Mg^{2+} and SO_4^{2-} growth inhibition data in Nielsen et al.[10].

Supplementary Table 1: Fit parameters for the extended *microkinetic model*

	K_{Mg}± std. dev.	K_{SO_4}± std. dev.	Residual
Mg^{2+}, SO_4^{2-}, $MgSO_4^0$	580 ± 43	683 ± 52	Total = 0.149

Because we do not know the detailed step structure on the growing crystals, we used the average energies for Ca^{2+} and CO_3^{2-} at both types of sites[11]. Thus, the derived adsorption energies for Mg^{2+} and SO_4^{2-} should also be interpreted to be average adsorption energies for the ions on the two types of steps, although the resulting value is clearly dominated by the weakest inhibition, as discussed in the main text.

Supplementary Table 2: Fit parameters for the *kink blocking model*

	$k_A = k_B$± std. dev.	$\nu_A = \nu_B$± std. dev.	k_M/ν_M± std. dev.	Resid. (total = 0.262)
Mg^{2+}	7181± *	0	668 ± 92	0.175
SO_4^{2-}	6E-3 ± 3E-2	0	400 ± 919	0.043
$MgSO_4^0$	5302 ± 3E+8	0 ± 3	1541 ± 5122	0.0442

* because of numerical instabilities, a standard deviation could not be obtained.

Supplementary Table 3: Fit parameters for the *incorporation inhibition model*

	k_{A}	k_{B}	k_{M}	$k_{\mathrm{B-M}}$	$\nu_{\mathrm{A}}/\nu_{\mathrm{B}}$	$\nu_{\mathrm{B-M}}/\nu_{\mathrm{M}}$	Resid. (total = 0.164)
Mg^{2+}	99	287	31	21	303	177	0.0648
SO_4^{2-}	336	198	10	4	52	253	0.0711
$MgSO_4^0$	209	204	0.465	0.0362	103	56	0.0279

With the simulated annealing method, standard deviations can not be extracted from the fitting algorithm.

In Supplementary Table 1-3, k_X denotes the ion attachment rate coefficient (in $s^{-1}M^{-1}$) and ν_{X} represents the detachment rate coefficient (in s^{-1}) of species X. $k_{\mathrm{B-M}}$ and $\nu_{\mathrm{B-M}}$ are the attachment/detachment rate coefficients for a crystal site next to an inhibitor site. K_X in the *microckinetic model* represents the adsorption energy equilibrium constant on a calcite step for species X.This can be M = Mg, A = Ca and B = CO_3 (first row in Supplementary Table 3) or M = SO_4, A = CO_3 and B =Ca (second row in Supplementary Table 3); M = $MgSO_4$, A = Ca and B = CO_3 (last row in Supplementary Table 3).

Supplementary Note 4
Inorganic ion pair formation in solution

Supplementary Figure 1 shows the difference in inhibition for Mg^{2+} and SO_4^{2-} using the extended *microkinetic model* with and without taking into account solution speciation. Without ion pair formation, the *microkinetic model* correctly predicts the behaviour of the systems but underestimates the inhibition effect slightly for Mg^{2+}. In this form, the *microkinetic model* and the *kink blocking model* perform equally well, which makes sense because both are adsorption models. The *microkinetic model* still uses only two parameters for two ions, compared with 9 parameters for the *kink blocking model* and it predicts the type of sites that must be blocked for inhibition to occur. The SO_4^{2-} data change very little upon including solution speciation, which is probably the reason why a Langmuir curve described the SO_4^{2-} data well in Nielsen et al[10]. When solution speciation is included, the fit for Mg is significantly improved, decreasing the residual for the combined Mg^{2+}, SO_4^{2-} and $MgSO_4$ fits from 0.241 to 0.149. From this, we conclude that ion pair

formation in solution is an important mechanism in inhibiting calcite growth and must be considered. The formation of ion pairs decreases calcite saturation and decreases the number of $CaCO_3^0$ growth units in solution. Ion pair formation accounts for ~10% of the total inhibitory effect that Mg^{2+} provides, at the highest Mg^{2+} concentrations in the experiments, i.e. 10 to 15 mM.

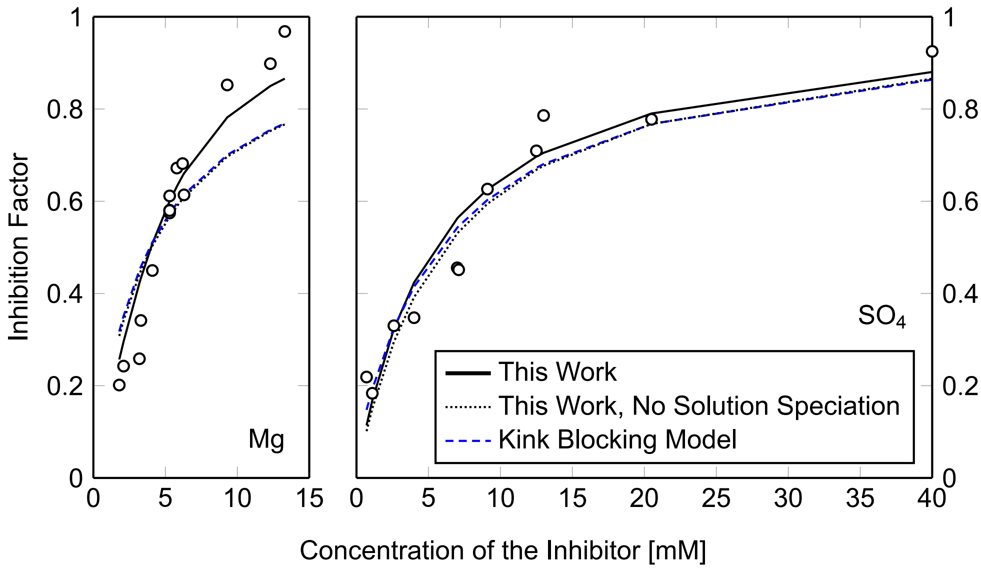

Supplementary Figure 1. Microkinetic model performance with and without considering solution speciation, compared with the kink blocking model.

Supplementary Note 5
Step blocking by organic acid adsorption

Supplementary Figure 2 presents the experimental data and predictions for the inhibition factor for the organic anions, acetate and benzoate in the absence of growth site blocking by adsorption, i.e. only solution complexing is taken into account. A weak but consistent underestimation of inhibition is observed, which in the case of 2 mM Ca + acetate amounts to ~20% for high inhibitor concentrations. Analogously to inhibition by Mg^{2+} in the previous section, reasonable agreement with experiments is obtained by including only the dominant growth inhibition mechanism but to obtain quantitative agreement with experiments, both adsorption and solution complexing must be accounted for in the calculations.

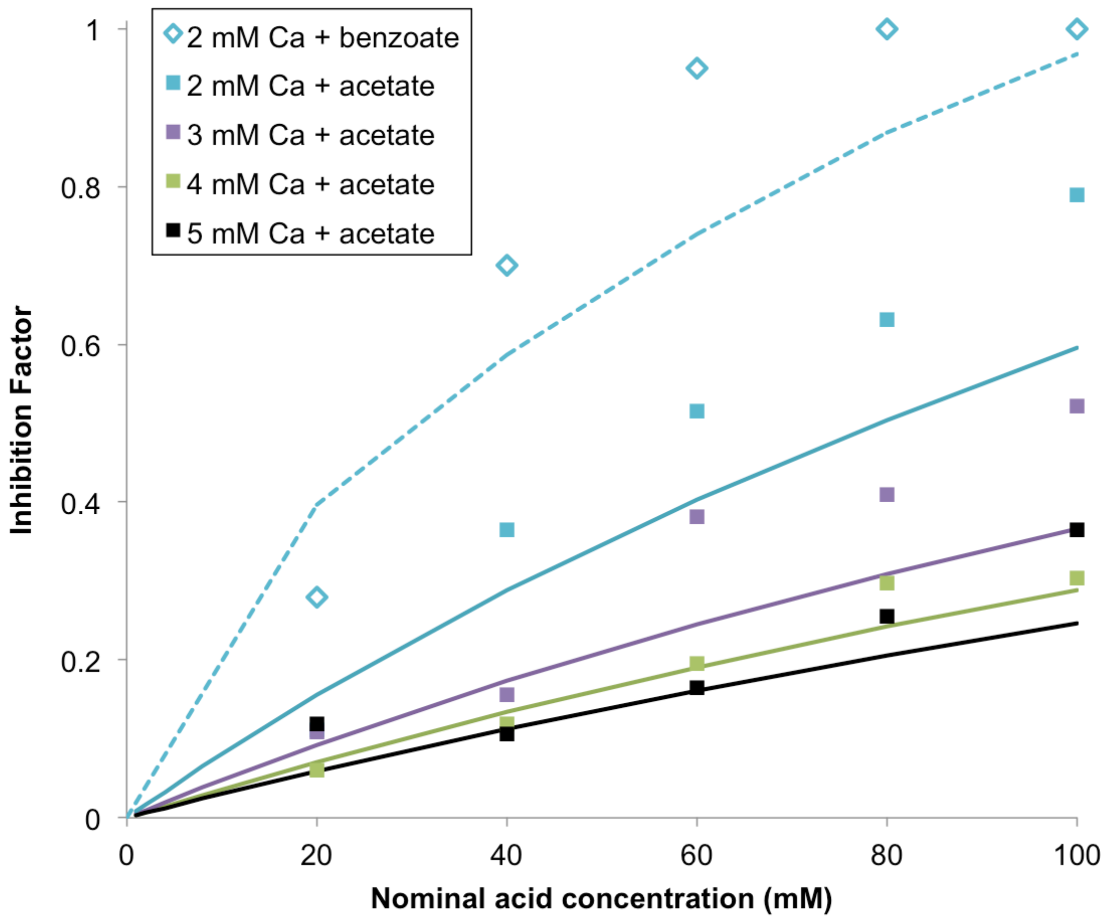

Supplementary Figure 2. The inhibition factor for calcite growth in the presence of the small carboxylate anions, acetate and benzoate, when only solution complexing was considered, in the absence of step adsorption. In the legend, x mM Ca refers to a starting solution of x mM $CaCl_2$ and x mM $NaHCO_3$.

Supplementary Note 6
Sensitivity analysis of the influence of adsorption energy on inhibition

To show how strongly the adsorption energy influenced inhibition, we plotted the inhibition index as a function of inhibitor concentration in Figure S3, for an inhibitor that would form solution complexes only to a minor degree, i.e. by artificially changing the SO_4^{2-} adsorption energy in the expressions above (Eqn S2a). Changing the free adsorption energy from -10 to -30 kJ/mol changes the effectiveness of the inhibitor tremendously because of the exponential dependence of the equilibrium constant for adsorption on the adsorption energy, $K_{\mathrm{ads}} = e^{-\frac{\Delta G_{\mathrm{ads}}}{RT}}$. Even very small changes in free adsorption energy have a very large impact on the effectiveness of the inhibitor. In this paper, we have not distinguished between the two types of calcite step (i.e.

obtuse and acute) because we are comparing with the macroscopic quantity. However, the high sensitivity of growth inhibition with even subtle changes in adsorption behaviour could very well explain the strong shape dependence of growth spirals on calcite because the adsorption energies would be different on the two types of steps[12,11]

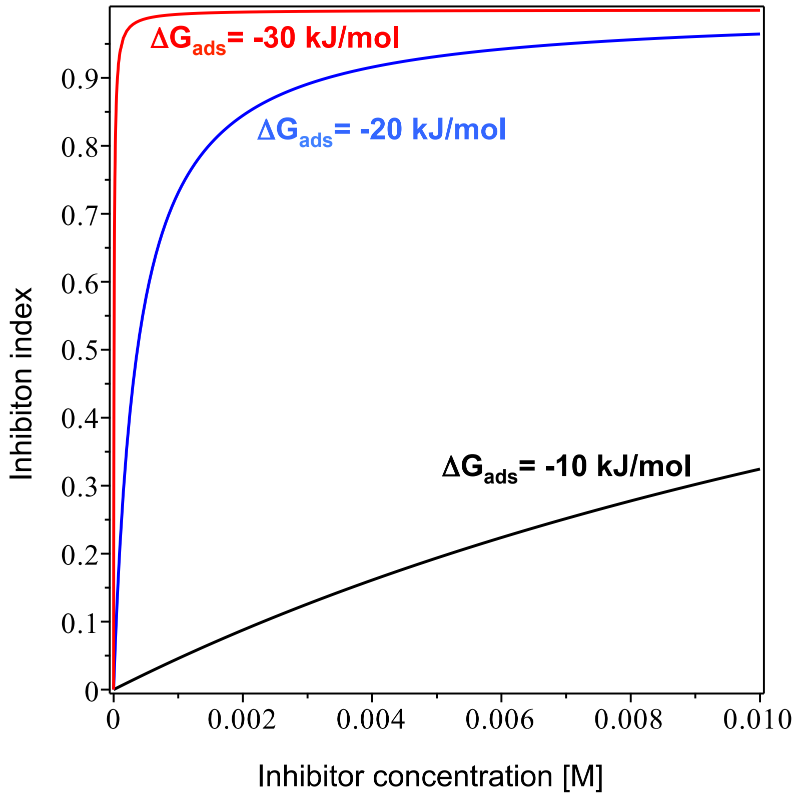

Supplementary Figure 3. Sensitivity analysis of the predicted inhibition index as a function of inhibitor concentration for three values of ΔG_{ads}, determined with Supplementary Equation 2.

The very strong dependence of inhibition capacity on adsorption energy also demonstrates one of our points in the main paper, that cation incorporation into the mineral surface (not simply adsorption) changes the energy landscape of the surface, thus adsorption energy for organic inhibitors. Only small changes in adsorption energy are required for large changes in growth rate and changes on the order 10 kJ/mol have been predicted[13]. This could potentially change inhibition capacity of organic inhibitors by about an order of magnitude, demonstrating the strong effect inorganic ion incorporation could have on organic inhibitors that are also present, if step blocking is a major inhibition mechanism.

Supplementary References

1 Cabrera, N. & Vermilyea, D. in *Growth and perfection of crystals; proceedings* (ed R. H. Doremus) (Wiley, 1958).
2 Nielsen, L. C., De Yoreo, J. J. & DePaolo, D. J. General model for calcite growth kinetics in the presence of impurity ions. *Geochim. Cosmochim. Acta* **115**, 100-114 (2013).
3 Potapenko, S. Y. Moving of step through impurity fence. *J. Cryst. Growth* **133**, 147-154 (1993).
4 Wang, L. J. *et al.* Constant composition studies verify the utility of the cabrera-vermilyea (C-V) model in explaining mechanisms of calcium oxalate monohydrate crystallization. *Cryst. Growth Des.* **6**, 1769-1775 (2006).
5 Weaver, M. L. *et al.* Improved model for inhibition of pathological mineralization based on citrate-calcium oxalate monohydrate interaction. *ChemPhysChem* **7**, 2081-2084 (2006).
6 Weaver, M. L. *et al.* Inhibition of calcium oxalate monohydrate growth by citrate and the effect of the background electrolyte. *J. Cryst. Growth* **306**, 135-145 (2007).
7 Weaver, M. L., Qiu, S. R., Friddle, R. W., Casey, W. H. & De Yoreo, J. J. How the overlapping time scales for peptide binding and terrace exposure lead to nonlinear step dynamics during growth of calcium oxalate monohydrate. *Cryst. Growth Des.* **10**, 2954-2959 (2010).
8 Lutsko, J. F. *et al.* Crystal growth cessation revisited: The physical basis of step pinning. *Cryst. Growth Des.* **14**, 6129-6134 (2014).
9 Zhang, J. W. & Nancollas, G. H. Kink density and rate of step movement during growth and dissolution of an AB crystal in a nonstoichiometric solution. *J. Colloid Interface Sci.* **200**, 131-145 (1998).
10 Nielsen, M. R. *et al.* Inhibition of calcite growth: Combined effects of Mg^{2+} and SO_4^{2-}. *Cryst. Growth Des.* **16**, 6199-6207 (2016).
11 Andersson, M. P. *et al.* A microkinetic model of calcite step growth. *Angew. Chem. Int. Ed.* **55**, 11086-11090 (2016).
12 Kristensen, R., Stipp, S. L. S. & Refson, K. Modeling steps and kinks on the surface of calcite. *J. Chem. Phys.* **121**, 8511-8523 (2004).
13 Andersson, M. P., Dideriksen, K., Sakuma, H. & Stipp, S. L. S. Modelling how incorporation of divalent cations affects calcite wettability-implications for biomineralisation and oil recovery. *Sci. Rep.* **6** (2016).
